# Mirabegron and antimuscarinics for treating ureteral stent-related symptoms: a systematic review and meta-analysis of RCTs

**DOI:** 10.3389/fphar.2023.1266636

**Published:** 2023-10-18

**Authors:** Youyi Lu, Qi Li, Qingsong Zou, Yuanshan Cui

**Affiliations:** ^1^ Department of Urology, The Affiliated Yantai Yuhuangding Hospital of Qingdao University, Yantai, Shandong, China; ^2^ Department of Endocrinology, Yantai Municipal Government Hospital, Yantai, Shandong, China

**Keywords:** mirabegron, antimuscarinics, ureteral stent-related symptoms, Ureteral Stent Symptom Questionnaire, meta-analysis

## Abstract

**Objective:** We conducted a meta-analysis to assess the efficacy and safety of mirabegron (50 mg/day) and antimuscarinics in treating ureteral stent-related symptoms (SRSs).

**Methods:** All randomized controlled trials (RCTs) were identified by searching PubMed, Embase, Web of Science, and Cochrane Library. The RevMan version 5.3.0 software was used for statistical analysis.

**Results:** This meta-analysis included five RCTs involving 317 patients. A fixed effects model revealed that mirabegron was superior to antimuscarinics in treating urinary symptoms (MD −1.39, 95% CI −2.63 to −0.15, *p* = 0.03) and general health (MD −1.65, 95% CI −2.60 to −0.69, *p* = 0.0007) 1 week after treatment initiation. We observed no significant differences in body pain (MD 0.05, 95% CI −1.06 to 1.15, *p* = 0.94), work performance (MD −0.86, 95% CI −1.77 to 0.06, *p* = 0.07), and sexual matters (MD 0.03, 95% CI −0.77 to 0.83, *p* = 0.94). Two weeks after treatment initiation, the ureteral stent symptom questionnaire (USSQ) revealed no significant differences between the two groups. The mirabegron group demonstrated a significant improvement in the quality of life (QoL) (MD −0.18, 95% CI −0.34 to −0.01, *p* = 0.03), while the International Prostate Symptom Score did not reveal a significant difference between the two groups (MD −0.74, 95% CI −1.79 to 0.32, *p* = 0.17). Regarding safety, a pooled data analysis presented that the incidence of constipation was lower in the mirabegron group (OR 0.10, 95% CI 0.01 to 0.77, *p* = 0.03). The mirabegron and antimuscarinics groups did not differ significantly concerning the risk of dry mouth (OR 0.15, 95% CI 0.02 to 1.27, *p* = 0.08).

**Conclusion:** Mirabegron is superior to antimuscarinics in alleviating ureteral SRSs and improving QoL. Additionally, mirabegron 50 mg/day presented safety with a lower incidence of constipation.

## 1 Introduction

With the rapid development of endourology, double J (DJ) stents have become widely used in many minimally invasive treatment surgeries. Ureteral stents are important in supporting the ureter and ureteral drainage and are primarily used to treat ureter obstruction and identify the ureter during pelvic surgery. However, the friction of the ureteral stents against the urinary tract may cause ureteral stent-related symptoms (SRSs), such as irritable bladder symptoms (urgent or frequent urination, dysuria, and hematuria) and stent-related body pain. SRSs reduce the life quality in up to 80% of the patients (De Coninck et al., 2020).

Although the mechanism is completely different, ureteral SRSs are similar to symptoms of overactive bladder (OAB). Therefore, the treatment for OAB is inferred to be effective in alleviating ureteral SRSs. Antimuscarinics, as typical pharmacotherapies for OAB treatment (Lightner et al., 2019), have proven beneficial in alleviating ureteral SRSs. The most commonly used antimuscarinics are oxybutynin, tolterodine, darifenacin, and solifenacin. However, adverse drug effects of antimuscarinics are common, such as dry mouth, blurred vision, constipation, and dyspepsia (Zhou et al., 2015). These adverse effects result in poor treatment compliance in patients.

Mirabegron, a β3 adrenergic receptor agonist, is recommended as an alternative treatment for SRSs with muscarine antagonist monotherapy (Sacomani et al., 2019). It significantly benefits the treatment of ureteral SRSs when compared to placebo or blank control (Li et al., 2022; Van Besien et al., 2022). A recent study has suggested that mirabegron has a favorable balance of efficacy and tolerability (Kelleher et al., 2018).

Although several studies have compared mirabegron with antimuscarinics for ureteral SRSs, the conclusions are inconsistent. Therefore, we performed a systematic review and meta-analysis based on randomized controlled trials (RCTs) to assess the efficacy and safety of mirabegron *versus* antimuscarinics in treating ureteral SRSs.

## 2 Methods

### 2.1 Search strategy and inclusion criteria

Two authors searched the databases PubMed, Embase, Web of Science, and Cochrane Library to identify all eligible studies published up to May 2023. The search terms were as follows: (mirabegron OR beta-3 agonist) AND (antimuscarinic OR muscarinic antagonists OR anticholinergic OR solifenacin OR tolterodine OR oxybutynin OR darifenacin OR fesoterodine OR propiverine OR trospium OR imidafenacin) AND (stent OR ureteral stent-related symptoms OR SRS). References of the included articles were also manually searched to identify other relevant articles. Language restrictions were not applied to the search.

The inclusion criteria were studies that

(a) focused on patients undergoing ureteral stent implantation;
(b) compared mirabegron with antimuscarinics in treating ureteral SRSs;
(c) evaluated all outcomes before removal of stents;
(d) had full text and analyzable data available; and
(e) involved randomized controlled trials.

**FIGURE 1:**
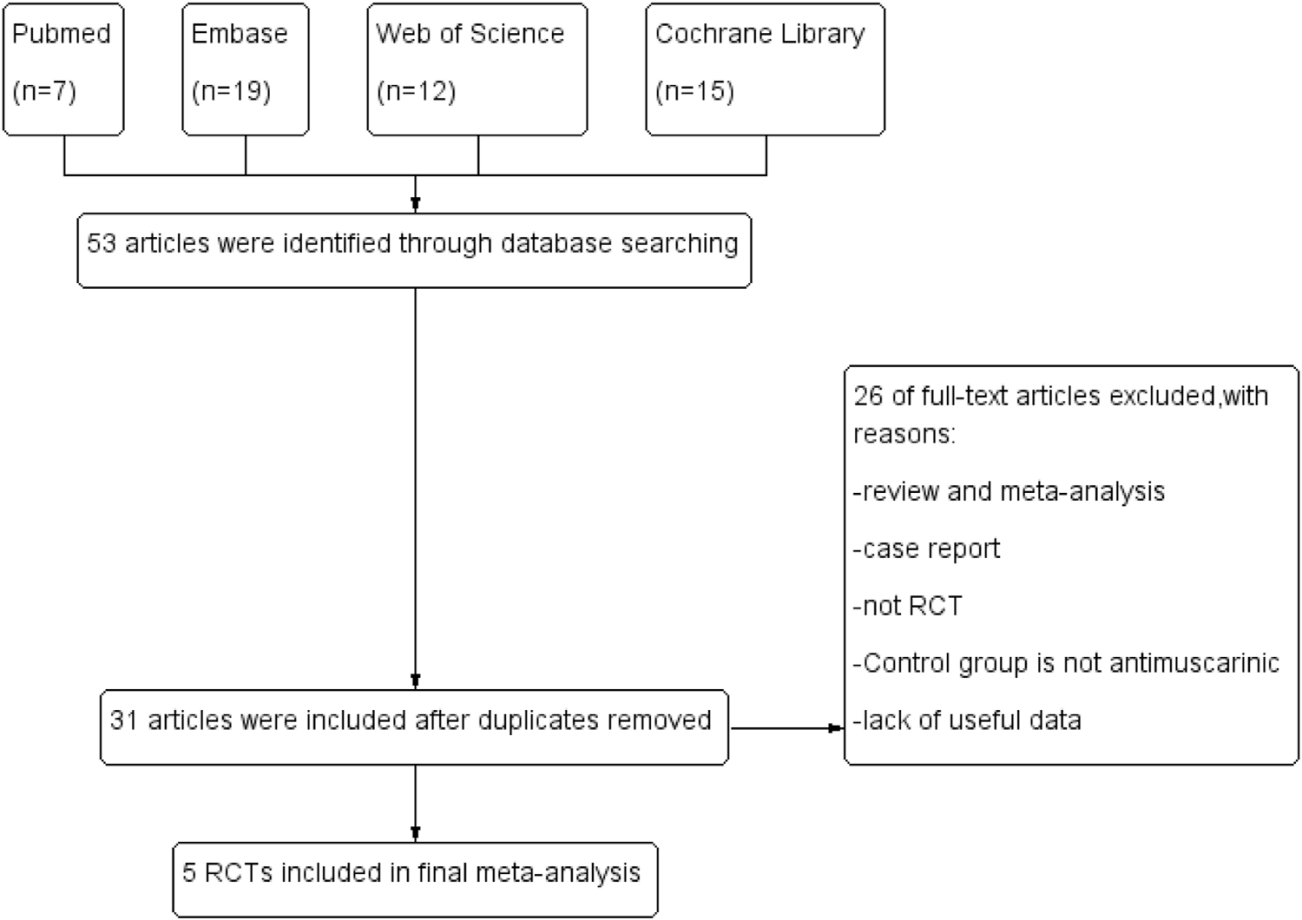
Flow diagram.

Two authors read all the searched articles separately and confirmed the final included articles by a consensus discussion. The flow diagram is presented in Figure 1.

### 2.2 Quality assessment

**FIGURE 2:**
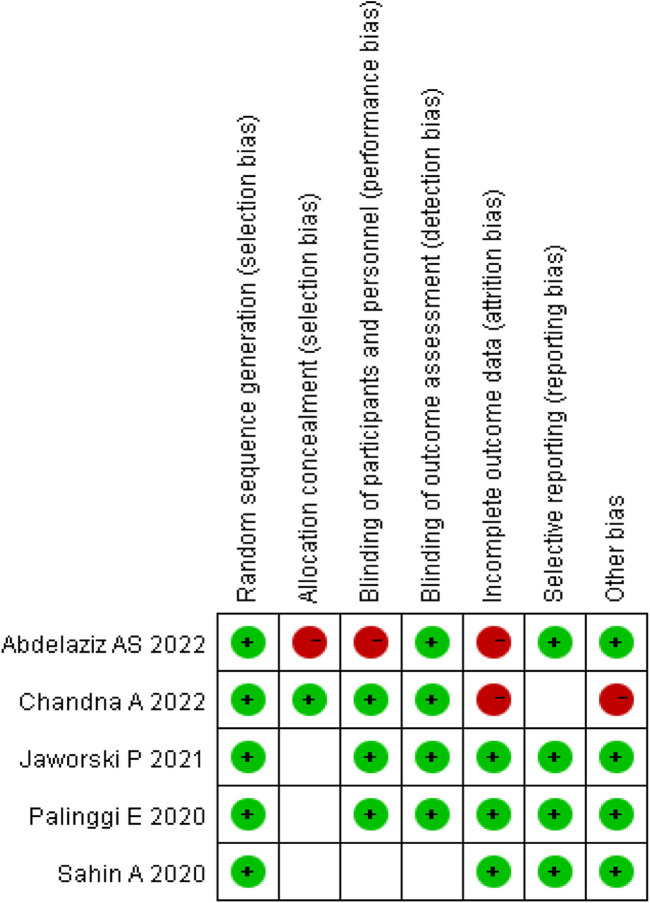
Risk of bias summary.

The quality of the included RCTs was assessed using the Cochrane Risk of Bias Tool. Each trial was evaluated on the basis of high, low, or unclear risk of bias (Higgins and Green, 2011). The risk of bias summary is illustrated in Figure 2.

### 2.3 Data extraction

Two authors independently collected the data. Controversial data were confirmed by consensus. The following characteristics of the included studies were collected: the primary author, publication year, country, intervention therapy, sample size, outcomes, inclusion population, and study design.

The study’s primary outcome was the ureteral stent symptom questionnaire (USSQ) (Joshi et al., 2003a). The secondary outcomes were the International Prostate Symptom Score (IPSS), quality of life (QoL), and adverse events (AEs). The variables were pooled for analysis only when evaluated by more than two studies.

### 2.4 Statistical analysis

The RevMan version 5.3.0 software (The Cochrane Collaboration, London, United Kingdom) (Higgins and Green, 2008) was used for statistical analysis. The mean difference (MD) with 95% confidence intervals (CIs) was used to assess the continuous data, while the odds ratio (OR) with 95% CIs was applied to assess the dichotomous data. A fixed effects model was used in homogenous studies (*p*-value of χ^2^ test ≥0.05 and I^2^ < 50%); otherwise, a random effects model was applied to heterogeneous studies (*p*-value of χ^2^ test <0.05 and I^2^ ≥ 50%). A *p*-value < 0.05 was considered statistically significant.

## 3 Results

### 3.1 Characteristics of eligible studies

We finally identified five RCTs (Palinggi et al., 2020; Sahin et al., 2020; Jaworski et al., 2021; Abdelaziz et al., 2022; Chandna et al., 2022) in this meta-analysis through database search: three RCTs compared the effects of mirabegron and solifenacin (Palinggi et al., 2020; Abdelaziz et al., 2022; Chandna et al., 2022); one compared the effects of mirabegron and oxybutynin (Jaworski et al., 2021); and another compared the effects of mirabegron and a combination of solifenacin and tamsulosin (Sahin et al., 2020). Table 1 lists the primary characteristics of the included studies.

**TABLE 1:** Main characteristics of the included studies.

Study	Country	Experimental group (M)	Control group (C)	Outcomes	Sample size		Inclusion population	Study design
					M	C		
Chandna et al. (2022)	India	Mirabegron (50 mg/day)	Solifenacin (5 mg/day)	USSQ	41	40	All patients above 18 years of age undergoing unilateral ureteral stent placement following ureteroscopic lithotripsy, percutaneous nephrolithotomy, or robot-assisted/laparoscopic pyeloplasty were included	Patients were followed up postoperatively at 10 days (first visit), 4 weeks (second visit), and subsequently, 2 weeks after stent removal
Jaworski et al. (2021)	Brazil	Mirabegron (50 mg/day)	Oxybutynin (5 mg/day)	USSQ	19	21	Inclusion criteria were to have had a ureteral stent inserted after a minimally invasive urinary stone treatment procedure (ureteroscopy, percutaneous nephrolithotripsy, or laparoscopy) or simple ureteral stent insertion without definite stone treatment for ureteral calculi	A Portuguese language-validated form of the USSQ was given to all participants through a phone call at three different moments (3rd, 6th, and 15th postoperative days)
				QoL				
Abdelaziz et al. (2022)	Egypt	Mirabegron (50 mg/day)	Solifenacin (5 mg/day)	USSQ	34	32	Patients 18–50 years old with an indication for endoscopic unilateral DJ stent insertion after ureteric stones and/or kidney stones treatment	Mirabegron/solifenacin started on the 4th day post-stent insertion, and USSQ and IPSS assessed on the day of stent removal (14 ± 2 days)
				IPSS				
				QoL				
Sahin et al. (2020)	Turkey	Mirabegron (50 mg/day)	Solifenacin (10 mg/day)/tamsulosin (0.4 mg/day)	VAPS	40	40	Patients with ureteral obstruction were included. Most of these patients had urinary stone disease and related interventions performed. However, in some of the patients, catheters were placed because of external factors that cause ureteral obstructions such as gynecological and colorectal malignancies	After 6 weeks, the VAPS, OAB-q index, and IPSS forms were replenished
				OAB-q				
				IPSS				
Palinggi et al. (2020)	Indonesia	Mirabegron (50 mg/day)	Solifenacin (5 mg/day)	USSQ	25	25	The inclusion criteria included patients aged 18–79 years who had undergone an endourology or a PCNL operation followed by unilateral DJ stent insertions. We also included patients with an indication of DJ stent with ureteric stones <10 mm (with or without pelvic dilatation, calyx, or ureters), ureteric stenosis, and/or kidney stones and who had undergone shockwave lithotripsy with DJ stent unilateral installation for the first time	The USSQ could be filled on the 7th day after surgery. Patients who showed symptoms of lower urinary complaint were given one of the treatments for 3 weeks, and the USSQ was conducted again on the 14th, 21st, and 28th day after surgery

USSQ, Ureteral Stent Symptom Questionnaire; IPSS, International Prostate Symptom Score; Qol, quality of life; OAB-q, Overactive Bladder questionnaire; VAPS, Visual Analogue Pain Scale; PCNL, percutaneous nephrolithotomy.

The meta-analysis included 317 participants [men = 188 (59.3%), women = 129 (40.7%)]. The basic clinical features between the two groups were similar regarding sex (OR 1.41, 95% CI 0.89 to 2.24, *p* = 0.14), age (MD −1.89, 95% CI −4.61 to 0.84, *p* = 0.17), and stent action (OR 1.15, 95% CI 0.66 to 2.01, *p* = 0.62), as shown in Table 2.

**TABLE 2:** Clinical characteristics.

Variable	N	I^2^ (%)	95% CI	*p*-Value
Sex (male/female)	5	**23**	**OR 1.41 (0.89, 2.24)**	**0.14**
Age (years)	4	**11**	**MD −1.89 (−4.61, 0.84)**	**0.17**
Action (Left/Right)	3	**0**	**OR 1.15 (0.66, 2.01)**	**0.62**

OR, odds ratio; MD, mean difference; CI, confidence interval. The bold value indicates in order to clarify whether there were statistical differences in clinical characteristics, and to exclude outcomes bias caused by them.

### 3.2 Efficacy

#### 3.2.1 Ureteral Stent Symptom Questionnaire

Four RCTs (Palinggi et al., 2020; Jaworski et al., 2021; Abdelaziz et al., 2022; Chandna et al., 2022) designed multiple follow-up visits on the USSQ to study SRSs. The measured time point of the questionnaire ranged from 3 days to 6 weeks. Finally, we analyzed all domain scores of the USSQ to compare mirabegron and antimuscarinics at 1 week and 2 weeks after initiating treatment.

**FIGURE 3:**
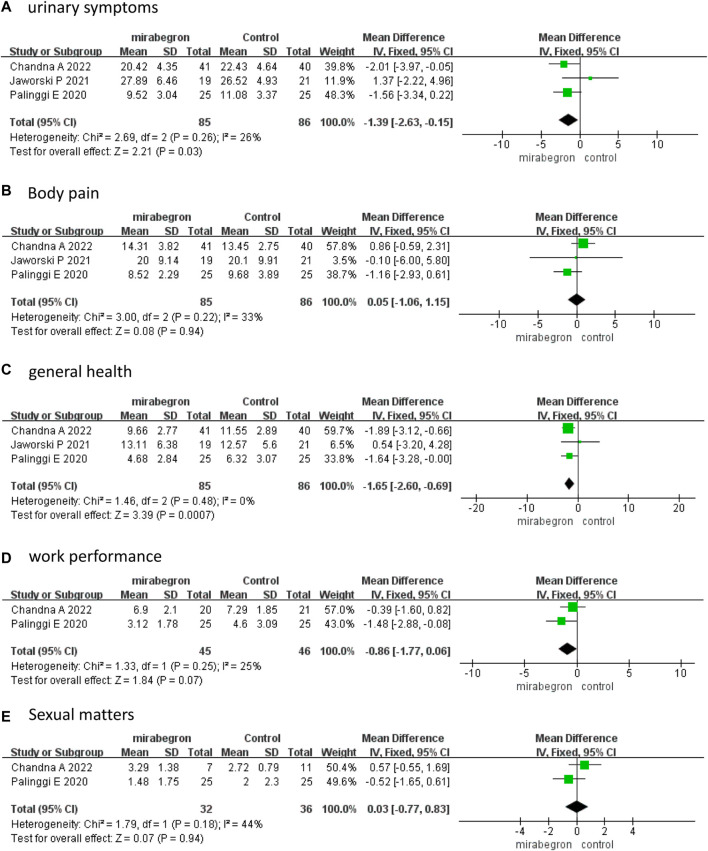
Forest plots comparing mirabegron with control for Ureteral Stent Symptom Questionnaire (USSQ) **(A)** urinary symptoms, **(B)** body pain, **(C)** general health, **(D)** work performance, and **(E)** sexual matters at 1 week.

Three studies (Palinggi et al., 2020; Jaworski et al., 2021; Chandna et al., 2022) that enrolled 171 participants (85 in the mirabegron group and 86 in the antimuscarinics group) analyzed the USSQ at week 1. A fixed effects model revealed that mirabegron significantly reduced urinary symptoms (MD −1.39, 95% CI −2.63 to −0.15, *p* = 0.03) and improved general health (MD −1.65, 95% CI −2.60 to −0.69, *p* = 0.0007) (Figures 3A, C). The USSQ body pain (MD 0.05, 95% CI −1.06 to 1.15, *p* = 0.94), work performance (MD −0.86, 95% CI −1.77 to 0.06, *p* = 0.07), and sexual matters (MD 0.03, 95% CI −0.77 to 0.83, *p* = 0.94) (Figures 3B, D, E) were not significantly different between the two groups.

**FIGURE 4:**
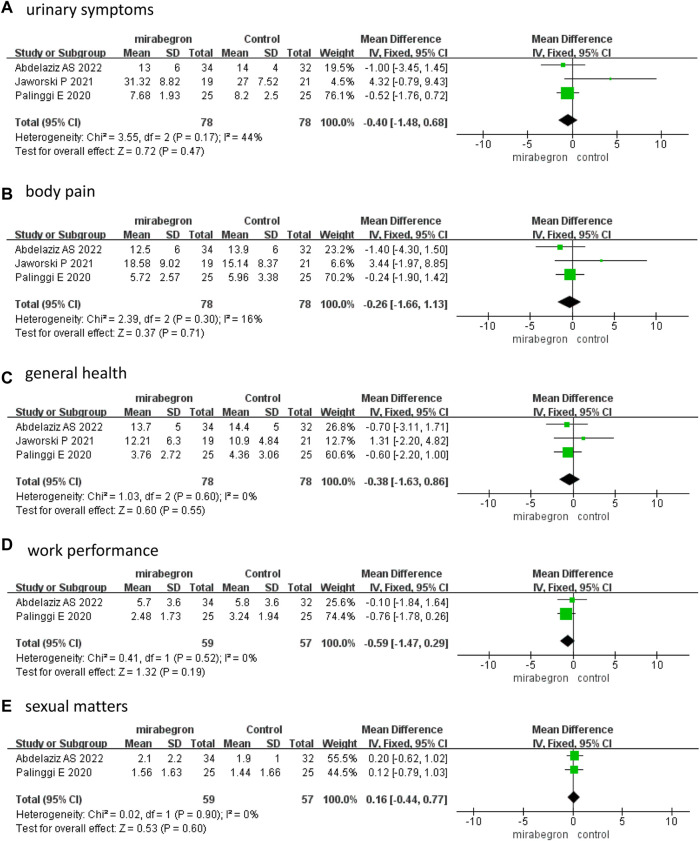
Forest plots comparing mirabegron with control for Ureteral Stent Symptom Questionnaire (USSQ) **(A)** urinary symptoms, **(B)** body pain, **(C)** general health, **(D)** work performance, and **(E)** sexual matters at 2 weeks.

The fixed effects model–pooled results indicated that there were no significant differences between the two groups in each domain score of the USSQ, which included urinary symptoms (MD −0.40, 95% CI −1.48 to 0.68, *p* = 0.47), body pain (MD −0.26, 95% CI −1.66 to 1.13, *p* = 0.71), general health (MD −0.38, 95% CI −1.63 to 0.86, *p* = 0.55), work performance (MD −0.59, 95% CI −1.47 to 0.29, *p* = 0.19), and sexual matters (MD 0.16, 95% CI −0.44 to 0.77, *p* = 0.60) (Figure 4) at 2 weeks.

#### 3.2.2 International Prostate Symptom Score

**FIGURE 5:**
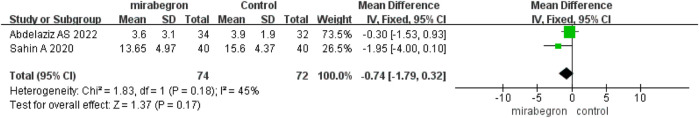
Forest plot comparing mirabegron with control for International Prostate Symptom Score (IPSS).

Abdelaziz et al. (2022) and Sahin et al. (2020) have reported on the IPSS; however, the follow-up times of the two articles differ (2 weeks and 6 weeks, respectively). The results exposed that the IPSS did not differ significantly between the mirabegron and antimuscarinic groups (MD −0.74, 95% CI −1.79 to 0.32, *p* = 0.17) (Figure 5).

#### 3.2.3 Quality of life

**FIGURE 6:**
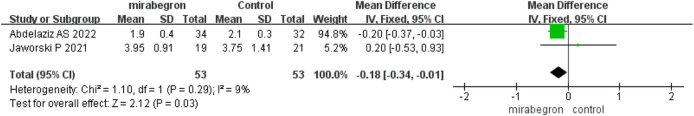
Forest plot comparing mirabegron with control for quality of life (QoL).

Jaworski et al. (2021) and Abdelaziz et al.( 2022) reported the QoL score and all the questionnaires were conducted at 2 weeks. The fixed effects model–pooled results disclosed that mirabegron significantly improved the QoL (MD −0.18, 95% CI −0.34 to −0.01, *p* = 0.03) (Figure 6).

### 3.3 Safety

Only two RCTs (Abdelaziz et al., 2022; Chandna et al., 2022) reported precise data on drug-related AEs. Chandna et al. (2022) reported three cases (two with headache and one with fatigue) in the mirabegron group, and nine (one headache, five constipation, and three dry mouth) cases in the antimuscarinics group, while Abdelaziz et al. (2022) reported five (three constipation and two dry mouth) cases in the antimuscarinics group. Our study observed no severe complications.

**FIGURE 7:**
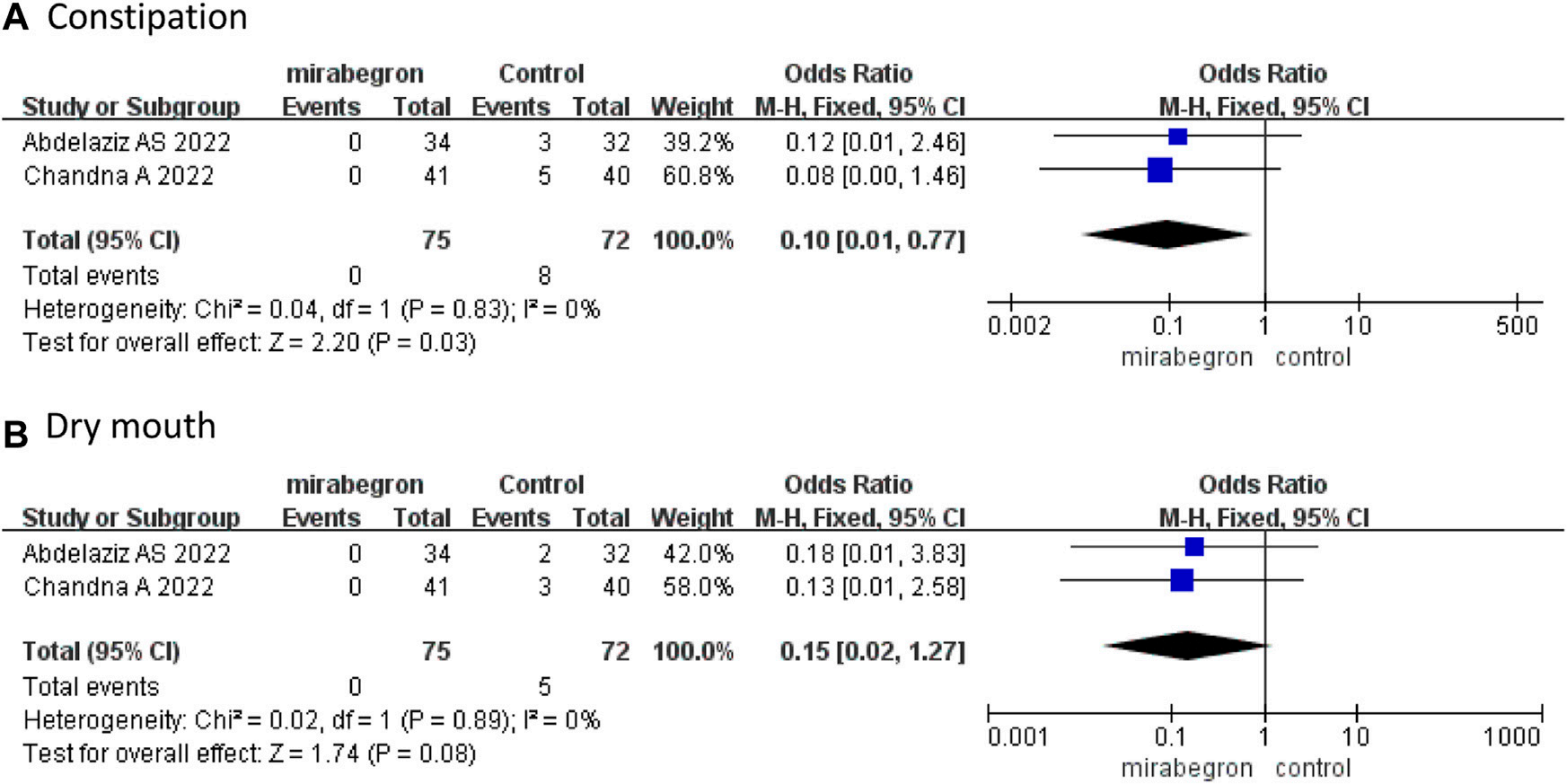
Forest plots comparing mirabegron with control for **(A)** constipation and **(B)** dry mouth.

The pooled results presented that the incidence of constipation was lower in the mirabegron group than in the antimuscarinics group (OR 0.10, 95% CI 0.01 to 0.77, *p* = 0.03) (Figure 7A). Dry mouth did not differ significantly between the mirabegron and antimuscarinics groups (OR 0.15, 95% CI 0.02 to 1.27, *p* = 0.08) (Figure 7B).

## 4 Discussion

Ureteral stents have been commonly used in urology, but consequent ureteral SRSs are also being increasingly reported. According to Joshi et al. (2003b), 80% of the patients suffered urinary symptoms and pain related to ureteral SRSs, 58% suffered reduced work performance, and 32% suffered sexual dysfunction. They developed a validated USSQ, which included urinary symptoms, body pain, general health, work performance, and sexual matters, to comprehensively evaluate ureteral SRSs and stent-related effects on the QoL. Currently, the USSQ is a reliable and robust tool for assessing SRSs. Additionally, IPSS, Overactive Bladder questionnaire (OAB-q), and Visual Analogue Pain Scale (VAPS) are also widely used to assess ureteral SRSs.

Van Besien et al. (2022) conducted a meta-analysis of mirabegron *versus* a placebo-controlled group in treating SRSs. Although only two RCTs were pooled for meta-analysis, the results showed a significant difference in favor of mirabegron in reducing USSQ urinary symptoms (*p* < 0.0001), IPSS (*p* = 0.006), and QoL (*p* = 0.0006). The pooled data analysis revealed that mirabegron improves USSQ general health (*p* = 0.04). Mirabegron and antimuscarinic drugs have been proven to alleviate SRSs, however, it is still unclear which drug is more effective. Therefore, we set antimuscarinic drugs as the control group and compared the efficacy and safety of mirabegron and antimuscarinic drugs in treating ureteral SRS. This is also the novelty of our meta-analysis. We only included RCTs to improve the quality of evidence. Moreover, included studies measured the outcomes 1 week and 2 weeks after medication separately. Therefore, we conducted a meta-analysis of the outcomes at different time points to understand the effect of mirabegron at different time points.

This meta-analysis revealed that mirabegron at 50 mg/day was superior to antimuscarinics in reducing the USSQ urinary symptoms and improving general health at the one-week visit. The other domains of the USSQ (body pain, work performance, and sexual matters) did not differ significantly. Reducing urinary symptoms in the early phase can improve patients’ satisfaction and compliance. Lower urinary symptoms, higher satisfaction, and fewer side effects may significantly decrease the general health score of the mirabegron group.

The pooled results of three RCTs (Abdelaziz et al., 2022; Jaworski et al., 2021; Palinggi et al., 2020) displayed no significant differences in all domain scores of the USSQ between the two groups at a two-week visit. Among them, two RCTs also reported the QoL score. The pooled results exhibited that the mirabegron group had a lower QoL score than the antimuscarinics group. However, we discovered that the QoL score and USSQ results at the two-week visit were inconsistent. Only two articles provided data for the QoL score, and the control group differed (oxybutynin and solifenacin, respectively). The differences in the control group may have contributed to the inconsistency of the QoL score and USSQ at the two-week visit. We believe that additional trials and better registration of the QoL can contribute to subgroup analysis and provide sufficient evidence. The IPSS was previously used for assessing patients with ureteral SRSs. In this meta-analysis, only two RCTs reported the IPSS. The pooled results presented that the IPSSs of the two groups were similar, but this appears inconsistent with the significant changes observed in urinary symptoms and the QoL. In our opinion, the number of included studies is relatively small, which may result in a bias of the IPSS. Besides, the control group of Sahin et al. (2020) was solifenacin + tamsulosin, and the combination effect may be stronger, adding to the bias. The difference in the follow-up time between the two RCTs (2 and 6 weeks, respectively) may also explain this inconsistency.

Our study’s pooled results of the USSQ urinary symptoms and general health differed at the 1- and 2-week follow-up. Jaworski et al. (2021), who compared the efficacy of mirabegron and oxybutynin in alleviating ureteral SRSs, also demonstrated the differences. They used a mixed linear model to conduct a longitudinal analysis for the USSQ at three follow-up visits (3rd, 6th, and 15th postoperative days) and discovered that the urinary symptoms' scores decreased over time. The decrease was particularly significant in the antimuscarinics group. Moreover, the time effect has also been proven in general health (*p* < 0.05). This finding coincidentally explains the differences in outcomes on urinary symptoms and general health of our study. Similarly, Liu et al. (2016) also observed that time played a certain role in symptom relief. However, it does not affect the comparison of the overall scores between the two groups at different follow-up times.

The mechanisms of mirabegron treating ureteral SRSs are not yet fully understood. Shen et al. (2017) proved via immunochemical analysis that all β adrenergic receptor subtypes are observed in the mucosa and muscular layers of the human ureter. These β adrenergic receptors are stimulated by β3 agonists such as mirabegron that lead to a relaxing effect and ureter dilation. The mechanical stimulation of the ureteral stent on the bladder mucosa can cause involuntary contractions, and mirabegron may function by inhibiting involuntary bladder contractions (Rossanese et al., 2015).

Regarding safety, the most reported drug-related AEs of mirabegron have been hypertension, headache, and dry mouth (Chapple et al., 2014). Many studies have revealed that the persistence and adherence rates of mirabegron are higher than those of antimuscarinics due to fewer AEs, such as dry mouth and constipation (Chapple et al., 2017; Yeowell et al., 2018; Song et al., 2021). Similarly, the pooled data analysis of the AEs presented that the incidence of constipation was lower in the mirabegron group, but there was no significant difference regarding dry mouth between the mirabegron and antimuscarinic groups. In our study, only one article reported headache and no hypertension in the mirabegron group.

This meta-analysis included only RCTs, and there is no significant heterogeneity among the individual studies. However, several limitations must still be mentioned. First, the number of included studies is limited. The meta-analysis did not have any unpublished data. Second, not all included studies used the USSQ, reducing analyzable data. Finally, follow-up at 1 week may not exclude similar symptoms caused by the surgical procedure. A two-week visit may allow for a more comprehensive evaluation of ureteral SRSs in patients on medication. However, considering that the patients suffer the most in the first week after stent insertion, mirabegron should be recommended as the preferred treatment for ureteral SRSs, especially for patients who have severe SRSs in the first few days or who require SRSs to be alleviated.

In conclusion, mirabegron is superior to antimuscarinics in alleviating ureteral SRSs and improving the QoL. Additionally, mirabegron at 50 mg/day presented safety with a lower incidence of constipation. In the future, more large-scale and high-quality RCTs are expected to provide stronger data in this field. Moreover, the monitoring of drug-related AEs is insufficient. We suggest that future studies should focus on AEs to evaluate them better.

## Data Availability

The original contributions presented in the study are included in the article/Supplementary Material; further inquiries can be directed to the corresponding authors.

## Abbreviations

SRSs, stent-related symptoms; RCTs, randomized controlled trials; DJ, double J; OAB, overactive bladder; USSQ, Ureteral Stent Symptom Questionnaire; IPSS, International Prostate Symptom Score; QoL, quality of life; AEs, adverse events; MD, mean difference; IV, intravenous; CIs, confidence intervals; OR, odds ratio; OAB-q, Overactive Bladder questionnaire; VAPS, Visual Analogue Pain Scale.
